# Editorial: Machine Learning for Quantitative Neuroimaging Analysis

**DOI:** 10.3389/fnins.2022.925819

**Published:** 2022-05-25

**Authors:** Yuankai Huo, Dakai Jin, Yudong Zhang, Dazhou Guo, Zhishun Wang

**Affiliations:** ^1^Department of Computer Science, Vanderbilt University, Nashville, TN, United States; ^2^Alibaba Group DAMO Academy, New York, NY, United States; ^3^School of Computing and Mathematical Sciences, University of Leicester, Leicester, United Kingdom; ^4^Department of Psychiatry, Columbia University, New York, NY, United States

**Keywords:** neuroimaging, machine learning, artificial intelligence (AI), brain, quantitative

## 1. Structural Neuroimaging

Our knowledge of the brain structure and the brain function has been rapidly leveraged from examining post-mortem specimens to *in vivo* inspection of living brains. Such an ability to examine the structure and function of the human brain in a detailed and noninvasive manner is largely realized by modern brain imaging techniques (i.e., neuroimaging). In general, neuroimaging can be categorized into two families: structural and functional imaging.

Structural neuroimaging (e.g., MRI and CT) typically refers to the imaging methods whose aims are to visualize, quantify and analyze anatomical properties of the brain structures. The structure imaging methods are extremely useful in clinical practice for detecting brain lesions and physical abnormalities. For example, the structural neuroimaging has been broadly used in detecting and quantifying brain tumors, bleeding, multiple sclerosis (MS), blood clots, traumatic brain injury (TBI) etc. Moreover, additional analytics can be performed to extract quantitative measurements from structural properties such as the volume of a given structure or the thickness of a given cortical surface area. In this RT, many advanced machine learning algorithms have been developed for structural neuroimaging analytics.

Wang et al. proposed a novel machine learning feature representation (Biorthogonal Wavelet Features) and a novel algorithm (Fitness-Scaled Adaptive Genetic Algorithm) for multiple sclerosis (MS) recognition. MS lesion characterization is an essential application of structural neuroimaging analyses. For methodological development, the bior4.4 wavelet was used to extract multiscale coefficients. Then, different wavelet features, optimized algorithms, and augmentation were comprehensively evaluated on MRI image cohorts.

Zhou et al. proposed a multimodal machine learning framework that combined the Boruta based feature selection and Multiple Kernel Learning (MKL) to functional neuroimaging analyses. Specifically, the multimodal features from structural and functional MRI and Diffusion Tensor Images (DTI) were integrated for the diagnosis of early adolescent Attention-deficit/hyperactivity disorder (ADHD). This study demonstrated that the structural and function signals from neuroimaging provided complementary information for discriminating ADHD from healthy children. The support vector machine (SVM) had been employed on a children cohort aged 9–10 years from the Adolescent Brain and Cognitive Development (ABCD) study. The results concluded a pioneer study to combine structural and functional MRI (including DTI) for early adolescents of the ABCD study.

## 2. Functional Neuroimaging

Functional imaging (e.g., fMRI, EEG, PET) is generally used for identifying brain functional regions and interpreting underlying brain procedures that are associated with specific behavioral and cognitive tasks. For example, the functional neuroimaging technologies can be used as a research tools in cognitive neuroscience, psychology, and psychiatry. In the past few years, machine learning technologies have been broadly used in the functional neuroimaging analyses to explore the hidden regularities behind the human brain activity.

Qi et al. developed a core EEG analytics approach, called SEOWADE, as a accessible and affordable brain-computer interface (BCI) systems to interpret brain signals. The key motivation of such a study was to achieve high patient classification performance using non-stationary and low signal-to-noise ratio (SNR) EEG signals. Different from the standard spatial filtering based approaches, this study investigated motor imagery signals via orthogonal wavelet decomposition. Moreover, channel-wise spectral filtering were conducted simultaneously with another L2-norm regularization to improve the discriminability and generalizability of EEG signals. The proposed SEOWADE method outperformed the benchmark approaches on various analyses from comprehensive experimental validations.

Chou et al. proposed an automatic classification method using fMRI Independent Component Analysis (ICA) classification and a deep Siamese neural network. Different from the canonical time-consuming manual observation, the proposed method presented a deep learning based approach to learn discriminative biomarkers based on a based on a deep Siamese network. The major advantages of this supervised design were that it required relatively fewer training data samples and without asking the specific ICA components. From cross-validation, the proposed approach can be used under a one-shot learning scenarios, which allowed the users to deploy the trained model on a new dataset by only seeing one example of each class. From experimental evaluations, the proposed method out-performed traditional convolutional neural networks (CNN) and template matching methods in identifying 11 subject-specific RSNs. Finally, The study demonstrated that the functional connectivity of default mode and salience networks was altered in a group analysis of mild traumatic brain injury (TBI), severe TBI, and healthy subjects.

Rakhimberdina et al. conducted a comprehensive review on deep learning based natural image reconstruction from fMRI. This survey paper presented an increasing prevalent topic, which is to decode visual information (reconstruction of the perceived natural images) from the human brain via fMRI. Specifically, the architectural design, benchmark cohorts, and evaluation metrics were systematically examined with standardized evaluation. Moreover, the strengths and limitations of such methods were also discussed with future potential directions.

## 3. Conclusion

This Research Topic promoted the development of advanced neuroimaging approaches for both structural and functional sides. Such methods are promising from comprehensive evaluations and can be further developed, evaluated, and translated into real clinical practice. In addition, an review article covered a wide range of topics and algorithms across the themes of this Research Topic. We hope this Research Topic is able to contribute to the theoretical foundation and clinical translation of using machine learning technologies in quantitative neuroimaging analyses.

## Author Contributions

All authors listed have made a substantial, direct, and intellectual contribution to the work and approved it for publication.

## Conflict of Interest

DJ and DG are employed by Alibaba Group (US) DAMO Academy. The remaining authors declare that the research was conducted in the absence of any commercial or financial relationships that could be construed as a potential conflict of interest.

## Publisher's Note

All claims expressed in this article are solely those of the authors and do not necessarily represent those of their affiliated organizations, or those of the publisher, the editors and the reviewers. Any product that may be evaluated in this article, or claim that may be made by its manufacturer, is not guaranteed or endorsed by the publisher.

